# Association of Carotid-Femoral Pulse Wave Velocity and Ejection Duration with Target Organ Damage

**DOI:** 10.31083/j.rcm2402041

**Published:** 2023-02-02

**Authors:** Yaya Bai, Huiying Jia, Alberto Avolio, Yi Qian, Junli Zuo

**Affiliations:** ^1^Department of Geriatrics, Ruijin Hospital, Shanghai Jiao Tong University School of Medicine, 200025 Shanghai, China; ^2^Department of Endocrine and Metabolic Diseases, Shanghai Institute of Endocrine and Metabolic Diseases, Ruijin Hospital, Shanghai Jiao Tong University School of Medicine, 200025 Shanghai, China; ^3^Department of Biomedical Sciences, Faculty of Medicine, Health and Human Sciences, Macquarie University, 2109 Sydney, Australia; ^4^Department of Cardiovascular Medicine, Ruijin Hospital, Shanghai Jiao Tong University School of Medicine, 200025 Shanghai, China

**Keywords:** carotid-femoral pulse wave velocity, ejection duration, target organ damage, renal damage, left ventricular hypertrophy

## Abstract

**Background:**

Carotid-femoral pulse wave velocity (cfPWV) and ejection duration (ED) have different impacts on target organ damage (TOD). The aim of this study was to determine the relationship of cfPWV and ED with TOD.

**Methods:**

A total of 1254 patients (64.27% males) from Ruijin Hospital were enrolled in this study from December 2018 to August 2022. Medical records, blood samples and urine samples were collected. The cfPWV was measured and ED was generated using SphygmoCor software (version 8.0, AtCor Medical, Sydney, Australia). TOD including left ventricular hypertrophy (LVH), microalbuminuria, chronic kidney disease (CKD), and abnormality of carotid intima-media thickness (CIMT) were evaluated.

**Results:**

Multiple stepwise linear regression models of cfPWV and ED (individually or together) showed that cfPWV was positively correlated with left ventricular mass index (LVMI) (β= 0.131, *p *= 0.002) and Log (albumin-creatinine ratio, ACR) (β= 0.123, *p *= 0.004), while ED was negatively correlated with LVMI (β= –0.244, *p <* 0.001) and positively correlated with the estimated glomerular filtration rate (eGFR) (β= 0.115, *p *= 0.003). When cfPWV and ED were added separately or together in multiple stepwise logistic regression models, cfPWV was associated with CKD [odds ratio (OR) = 1.240, 95% confidence interval (CI) 1.055–1.458, *p *= 0.009], while ED was associated with LVH (OR = 0.983, 95% CI 0.975–0.992, *p <* 0.001). In the control group with normal cfPWV and normal ED, LVH was significantly lower in patients with high ED (OR = 0.574, 95% CI 0.374–0.882, *p *= 0.011), but significantly elevated in those with high cfPWV and low ED (OR = 6.799, 95% CI 1.305–35.427, *p *= 0.023).

**Conclusions:**

cfPWV was more strongly associated with renal damage, while ED was more strongly associated with cardiac dysfunction. cfPWV and ED affect each other, and together have an effect on LVH.

## 1. Introduction

Carotid-femoral pulse wave velocity (cfPWV) is a gold standard measure of arterial stiffness. cfPWV is associated with target organ damage (TOD) such as left ventricular hypertrophy (LVH), chronic kidney disease (CKD), microalbuminuria, abnormality in carotid intima-medium thickness (CIMT), as well as cardiovascular events [1, 2, 3].

Ejection duration (ED) is defined as the time interval from opening to closure of the aortic valve [4], and is closely related to cardiac physiology and function [5]. The methods used to measure ED have changed over the years [6, 7, 8]. The main factor that shortens left ventricular ejection time (LVET) is the relative lengthening of the pre-ejection period (PEP), thereby delaying the onset of ejection. Further shortening of the LVET is associated with a decrease in stroke volume [4]. ED is associated with impairment of cardiac function and is a strong predictor of cardiovascular outcomes in certain patients, including those with hypertension [9], heart failure [10, 11], or other ischemic cardiac diseases [12, 13]. It has also been shown that ED is an independent predictor of incident heart failure [14]. When the arterial elastic modulus is constant, LVET has a dominant effect on the calculated PWV compared to the heart rate (HR) and to peripheral resistance [15]. Moreover, LVET but not HR is independently correlated with aortic PWV [16]. However, clinical applications of ED measurement in the general population that is free of cardiac diseases remains unclear, and the interaction of ED and/or cfPWV with TOD and cardiovascular events requires further investigation.

Therefore, in the present study we analyzed the associations of cfPWV and/or ED with TOD. This allowed exploration of the interaction between arterial stiffness and LVET, with the long-term goal of achieving individualized clinical management of ED and cfPWV in general patients.

## 2. Materials and Methods

### 2.1 Study Population

**Fig. 1.**
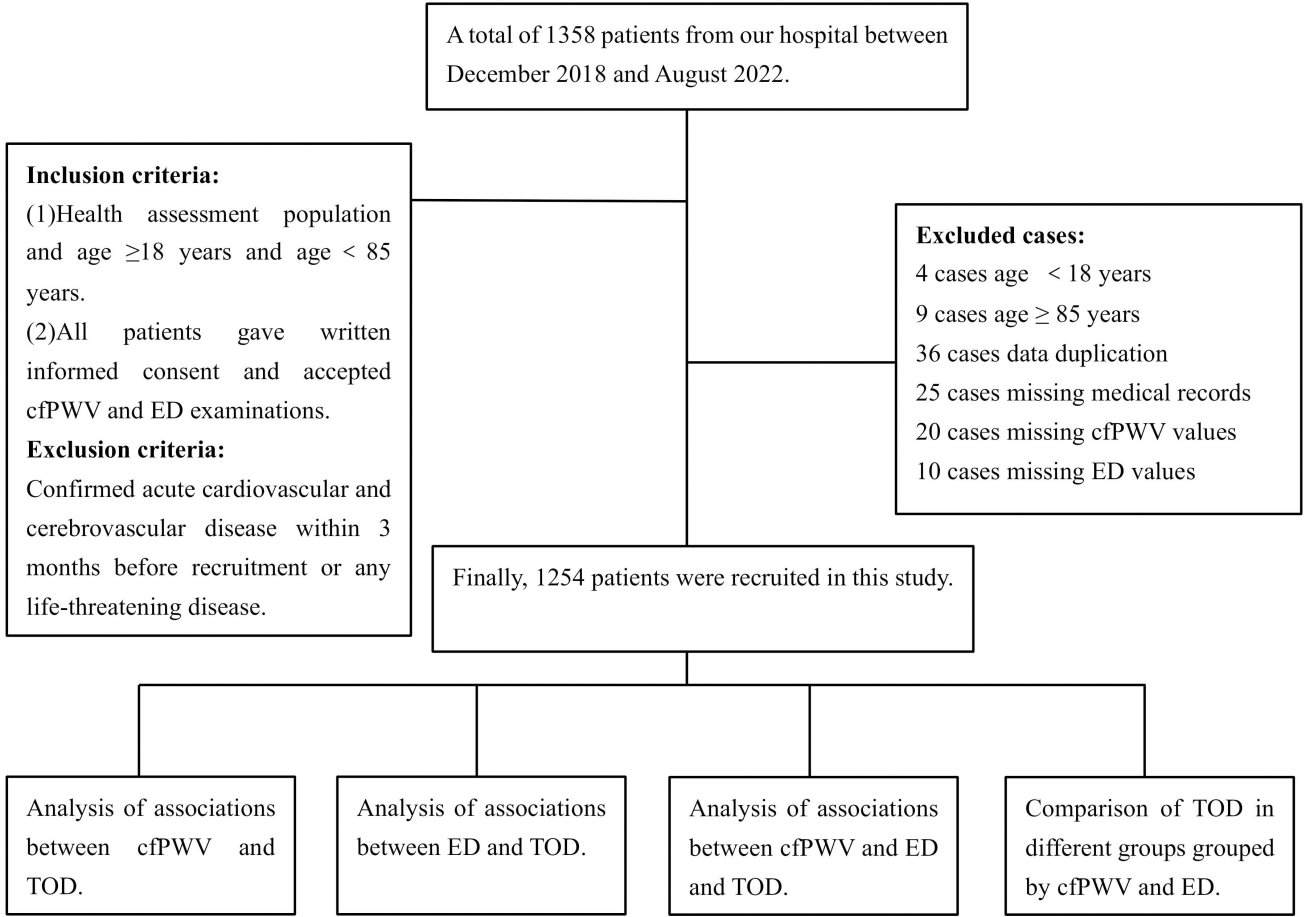
**Flowchart of the research protocol**.

A total of 1358 subjects who attended the Ruijin Hospital (affiliated with the Shanghai Jiao Tong University School of Medicine) from December 2018 to August 2022 were included in this study. The inclusion criteria were health assessment, age ≥18 years, and age <85 years. Written informed consent was given by all patients and all agreed to undergo cfPWV and ED examinations. Exclusion criteria included confirmed acute cardiovascular and cerebrovascular disease within 3 months of recruitment, any life-threatening disease such as hemorrhagic or ischemic stroke, severe arrhythmia, severe heart failure (New York Heart Association Class IV), acute coronary syndrome, and malignant tumor with a life expectancy of <5 years. Among the 1358 patients, 4 cases were excluded due to age <18 years and 9 cases due to age ≥85 years. In addition, 36 cases were excluded because of duplication of clinical data, 25 cases due to missing medical records, 20 cases because of missing cfPWV values, and 10 cases due to lack of ED values. This resulted in a final study cohort of 1254 patients. These were grouped according to cfPWV and ED values as follows: Group (control), Group (low ED), Group (high ED), Group (high cfPWV), Group (high cfPWV, low ED), Group (high cfPWV, high ED). “High cfPWV” was a cfPWV >10 m/s , while “normal cfPWV” was cfPWV ≤10 m/s. One of the high-risk factors for asymptomatic hypertensive TOD is cfPWV >10 m/s [17]. ED in the range of 281 ms to 321 ms was defined as “normal ED”, while ED <281 ms was defined as “low ED” and ED >321 ms as “high ED”. Clinical data including sex, age, height, body mass index (BMI), antihypertensive drugs (yes or no) and smoking history (yes or no) was collected using a standardized questionnaire. BMI was calculated as the ratio of body weight (kilograms) divided by the square of body height (meters). Venous blood and urine samples were collected after obtaining informed consent. Serum uric acid (UA), creatinine (Cr), triglyceride and total cholesterol (TC), low-density lipoprotein cholesterol (LDL-c), high-density lipoprotein cholesterol (HDL-c), fasting blood glucose (FBG) and hemoglobinA1c (HbA1c) were measured in the venous blood sample using standard methods. The urine sample was used to measure urinary albumin and creatinine. The research protocol for this study (Fig. 1) was approved by the Ethics Committee of Ruijin Hospital, Shanghai Jiao Tong University School of Medicine (Ethics No. 2011-30).

### 2.2 Measurement and Pulse Wave Analysis

A high-fidelity SPT-304 micromanometer (20172216993, Millar Instruments, Houston, TX, USA) interfaced with a laptop computer was used to obtain radial waveforms and pulse wave analysis measurements by applanation tonometry. SphygmoCor software (version 8.0, AtCor Medical, Sydney, Australia) was used to generate a reconstructed aortic pulse waveform from radial waveforms using a transfer function [18]. ED, subendocardial viability ratio (SEVR) and other hemodynamic indices including the central augmentation index (cAIx), cAIx adjusted to a heart rate of 75 bpm (beats per minute) (AIx@HR75), central diastolic blood pressure (cDBP), central systolic blood pressure (cSBP) and central mean arterial blood pressure (cMAP) were derived from the reconstructed aortic waveform. For calibrating radial waveforms, triplicate recordings of left brachial blood pressure and a 10-s sample of brachial pulse waves were measured by a validated Omron 705 CP oscillometric device (HEM-705cp, Omron, Kyoto, Japan) [19]. For this measurement, the subject was in the supine position in a quiet room with stable temperature for at least 10 minutes of rest, and without caffeine, smoking or exercise for 30 minutes prior to examination [20]. ED was reported in milliseconds (ED ms) and as a percentage of the cardiac cycle (ED%). It was defined as beginning with the initial upstroke of the forward wave and ending with occurrence of the dicrotic notch [21]. Peripheral mean arterial blood pressure (pMAP) was calculated using the following formula: pMAP = peripheral diastolic blood pressure (pDBP) + 1/3 [peripheral systolic blood pressure (pSBP) – pDBP]. Recordings were discarded if the diastolic or systolic variability of consecutive waveforms exceeded 5%, or if the raw amplitude of the recorded pulse wave signal was <80 mV. All recordings entered into the software package met the manufacturer’s quality control standards.

### 2.3 Carotid-Femoral Pulse Wave Velocity 

The carotid-femoral pulse wave velocity (cfPWV) was calculated using the formular: cfPWV (m/s) = [(the distance of the suprasternal notch to the femoral artery — the distance from the suprasternal notch to the carotid artery) (m)/the transit time of the pulse wave (s)]. Shortly after the measurement of office blood pressure, the right side carotid and femoral arterial waveforms were derived by applanation tonometry. Patients fasted overnight and no caffeine beverage or smoking was allowed within 3 hours of the measurement. PWV was measured using SphygmoCor (version 8.0, AtCor Medical, Sydney, Australia). For this study, “normal cfPWV” was defined as cfPWV ≤10 m/s, and “high cfPWV” as cfPWV >10 m/s.

### 2.4 Target Organ Damage (TOD)

#### 2.4.1 Left Ventricular Hypertrophy (LVH)

LVH was defined as a left ventricular mass index (LVMI) ≥115 g/$m^{2}$ in men and ≥95 g/$m^{2}$ in women. It was calculated using echocardiography and performed according to a standardized reading protocol. All indices were estimated by an experienced sonographer or cardiologist and were based on recommendations of the American Society of Echocardiography [22].

#### 2.4.2 Renal Abnormality

Spot morning urine samples obtained from participants were used to measure the urinary albumin-creatinine ratio (ACR). Abnormal albuminuria was defined as urine ACR >2.5 mg/mmol in males and >3.5 mg/mmol in females. As recommended in the Kidney Disease Outcomes Quality Initiative (K/DOQI) guidelines, the definition and diagnostic criteria for chronic kidney disease (CKD) was estimated glomerular filtration rate (eGFR) <60 mL/min/1.73 $m^{2}$, as calculated by the Modification of Diet in Renal Disease (MDRD) formula [23].

#### 2.4.3 Carotid Intima-Media Thickness (CIMT)

Carotid intima-media thickness was assessed bilaterally by high-resolution Doppler ultrasound (HD11EX Ultrasound, Philips Medical Systems, Andover, MA, USA) with a broadband linear array transducer, preferentially at frequencies >7 MHz. Intima-Media Thickness (IMT) was measured within a plaque-free region [24], preferably on the far wall of the common carotid artery and at least 5 mm below its end [25]. The average value of the three recordings measured separately at both the left and right carotid arteries during the diastolic portion of the cardiac cycle was calculated for each side. The average of the left CIMT and right CIMT [(Left CIMT + Right CIMT)/2] was calculated as the final CIMT. Plaques are focal structures that encroach into the arterial lumen by at least 0.5 mm or 50% of the surrounding IMT value, or show a thickness of >1.5 mm as measured from the intima-lumen interface to the media-adventitia interface [26]. A CIMT ≥0.9 mm and/or the presence of carotid plaques were defined as CIMT abnormality.

### 2.5 Statistics Analysis

All analyses were performed using SPSS 24.0 for Windows (SPSS Inc, Chicago, IL, USA). A two-sided *p* value of < 0.05 was considered statistically significant. The distribution for normality of quantitative parameters was checked by nonparametric One Sample K-S test, with *p >* 0.05 demonstrating the variable fits a normal distribution. Qualitative parameters were presented as numbers with the percentage in parentheses, and quantitative parameters as the mean ± standard deviation. These were compared between genders by the chi-squared test and by the two-independent sample student’s test, respectively. Correlations of cfPWV and ED with TOD were investigated by Pearson’s correlation analysis. Multivariate stepwise linear or logistic regressions analyses [forward likelihood ratio (LR)] were performed to evaluate the association of risk factors with TOD. cfPWV and ED were included either separately or together in the regression models, and in different groups classified according to cfPWV and ED values. Adjustment was made for covariates including sex, age, height, BMI, smoking history, antihypertensive drugs (yes or no), HDL-c, LDL-c, FBG, heart rate (HR), and pMAP. Only variables that remained statistically significant in the final model were presented.

## 3. Results

### 3.1 Baseline Clinical Characteristics of the Study Population

A total of 1254 patients (mean age 53.13 ± 12.62 years, 64.27% males) were recruited to this study. Males were significantly taller with larger BMI and higher incidence of smoking history and antihypertensive treatment compared to women (*p <* 0.05), but were significantly younger (*p <* 0.001). Women had significantly higher levels of TC, HDL-c and FBG than men (*p <* 0.05), but lower levels of UA and total triglycerides (TG) (*p <* 0.05). FBG and HbA1c levels were not significantly different between males and females (*p *> 0.05). For the peripheral and central hemodynamic indices, males had significantly higher peripheral diastolic blood pressure (pDBP), pMAP, cDBP, cMAP, cfPWV and SEVR than females (*p <* 0.05), whereas peripheral systolic blood pressure (pSBP) and cSBP were not significantly different between the two genders (*p *> 0.05). Moreover, females had significantly higher values for central augmentation pressure (cAP), cAIx, AIx@HR75 and ED (*p <* 0.05). Because urinary ACR was skewed, Log ACR was used in the logistic regression analysis. The values for LVMI, eGFR, CIMT and the percentage of CIMT abnormality were all significantly higher in men (*p <* 0.05), whereas the percentages for LVH and CKD were higher in women (*p <* 0.05) (Table 1).

**Table 1.** **Baseline clinical characteristics**.

Variable	Overall	Male	Female	*p* value
	N = 1254	N = 806	N = 448	
Age (years)	53.13 ± 12.62	52.11 ± 12.55	54.95 ± 12.57	<0.001
Sex, n (%)	NA	806/1254 (64.27)	448/1254 (35.73)	NA
Height (cm)	167.41 ± 8.18	171.41 ± 6.43	160.22 ± 5.69	<0.001
BMI (Kg/$m^{2}$)	25.32 ± 3.90	25.93 ± 3.75	24.23 ± 3.93	<0.001
Smoking history, n (%)	215/1254 (17.15)	199/806 (24.69)	16/448 (3.57)	<0.001
Antihypertensive agents, n (%)	391/1254 (31.18)	291/806 (36.10)	100/448 (22.32)	<0.001
Serum uric acid (μmol/L)	365.19 ± 96.41	394.99 ± 91.01	311.68 ± 81.66	<0.001
TG (mmol/L)	1.93 ± 1.62	2.09 ± 1.83	1.64 ± 1.08	<0.001
TC (mmol/L)	4.81 ± 1.08	4.71 ± 1.09	4.99 ± 1.02	<0.001
HDL-c (mmol/L)	1.15 ± 0.35	1.08 ± 0.35	1.27 ± 0.33	<0.001
LDL-c (mmol/L)	3.13 ± 0.80	3.08 ± 0.81	3.22 ± 0.78	0.005
FBG (mmol/L)	5.78 ± 1.77	5.85 ± 1.75	5.65 ± 1.80	0.055
HbA1c (%)	6.16 ± 1.21	6.17 ± 1.24	6.12 ± 1.17	0.556
pSBP (mmHg)	130.68 ± 18.57	131.45 ± 17.38	129.28 ± 20.48	0.058
pDBP (mmHg)	76.71 ± 11.97	78.14 ± 11.50	74.14 ± 12.36	<0.001
pMAP (mmHg)	94.70 ± 13.07	95.91 ± 12.41	92.52 ± 13.93	<0.001
cSBP (mmHg)	119.68 ± 17.86	119.95 ± 16.78	119.19 ± 19.66	0.492
cDBP (mmHg)	77.81 ± 12.10	79.23 ± 11.64	75.26 ± 12.50	<0.001
cMAP (mmHg)	95.52 ± 13.91	96.53 ± 13.19	93.71 ± 14.96	0.001
cAP	12.61 ± 7.52	11.58 ± 7.28	14.46 ± 7.62	<0.001
cAIX	28.81 ± 13.12	27.13 ± 13.25	31.83 ± 12.34	<0.001
cAIX@HR75	25.31 ± 11.83	23.54 ± 12.07	28.49 ± 10.70	<0.001
HR (beat/min)	69.24 ± 10.39	69.20 ± 10.24	69.32 ± 10.68	0.837
cfPWV (m/s)	8.24 ± 2.02	8.35 ± 1.96	8.05 ± 2.10	0.012
LVMI (g/$m^{2}$)	103.44 ± 26.37	106.45 ± 25.84	96.88 ± 26.35	<0.001
eGFR (mL/min/1.73 $m^{2}$)	90.28 ± 16.84	92.44 ± 17.23	86.39 ± 15.37	<0.001
LogACR (mg/mmol)	0.39 ± 0.36	0.39 ± 0.40	0.38 ± 0.25	0.685
CIMT (mm)	0.74 ± 0.14	0.75 ± 0.15	0.73 ± 0.12	0.036
LVH, n (%)	298/836 (35.65)	173/573 (30.19)	125/263 (47.53)	<0.001
CKD, n (%)	36/1191 (3.02)	17/765 (2.22)	19/426 (4.46)	0.031
ACR abnormality, n (%)	129/754 (17.11)	94/520 (18.08)	35/234 (14.96)	0.293
CIMT abnormality, n (%)	386/791 (48.80)	270/524 (51.53)	116/267 (43.45)	0.032
ED (ms)	318.68 ± 26.11	314.83 ± 25.73	325.62 ± 25.37	<0.001
SEVR (%)	144.72 ± 25.85	148.89 ± 26.32	137.21 ± 23.19	<0.001

Data shown are the mean ± SD or as stated. *p* value: independent *t*-test for numeric variables and chi-square test for categorical variables. BMI, body mass index; TG, total triglycerides; TC, total cholesterol; HDL-c, high-density lipoprotein cholesterol; LDL-c, low-density lipoprotein cholesterol; FBG, fasting blood glucose; pSBP, peripheral systolic blood pressure; pDBP, peripheral diastolic blood pressure; pMAP, peripheral mean arterial pressure; cSBP, central systolic blood pressure; cDBP, central diastolic blood pressure; cMAP, central mean arterial pressure; cAP, central augmentation pressure; cAIx, central augmentation index; cAIx@HR75, cAIx adjusted to the heart rate of 75 bpm; HR, heart rate; cfPWV, carotid-femoral pulse wave velocity; eGFR, estimated glomerular filtration rate; LVMI, left ventricular myopathy index; CIMT, carotid intima-media thickness; ACR, albumin–creatinine ratio; LVH, left ventricular hypertrophy; CKD, chronic kidney disease; ED, ejection duration; SEVR, subendocardial viability ratio; NA, none.

### 3.2 Pearson Correlations of cfPWV and ED with TOD

Pearson correlation analysis showed that cfPWV was positively correlated with LVMI (r = 0.325, *p*< 0.001), LogACR (r = 0.188, *p <* 0.001) and CIMT (r = 0.283,* p <* 0.001), but negatively correlated with eGFR (r = –0.234, *p <* 0.001). ED was negatively correlated with LVMI (r = –0.132, *p <* 0.001), but showed no significant correlations with eGFR (*p = *0.118), LogACR (*p *= 0.184) or CIMT (*p =* 0.672) (Table 2).

**Table 2.** **Pearson correlations of cfPWV and ED with target organ damage**.

Variable	LVMI		eGFR		LogACR		CIMT	
	r	*p*	r	*p*	r	*p*	r	*p*
cfPWV	0.325**	<0.001	–0.234	<0.001	0.188**	<0.001	0.283**	<0.001
ED	–0.132**	<0.001	–0.045	0.118	–0.052	0.184	–0.015	0.672

cfPWV, carotid-femoral pulse wave velocity; ED, ejection duration; LVMI, left ventricular myopathy index; eGFR, estimated glomerular filtration rate; ACR, albumin–creatinine ratio; CIMT, carotid intima-media thickness. **, Significant at 0.01 level (two tailed).

### 3.3 Multivariate Stepwise Linear Regression Analysis for the Association of cfPWV and ED with Risk Factors 

cfPWV and ED were added separately into multiple stepwise linear regression models with risk factors. The results of this analysis showed that cfPWV was significantly associated with age, pMAP, FBG, HR, height, antihypertensive treatment (yes or no) and BMI (*p <* 0.05), while ED was significantly associated with sex, BMI, HR, HDL-c, pMAP and FBG (*p <* 0.05) (Table 3).

**Table 3.** **Multiple stepwise linear regression analysis of cfPWV and ED with risk factors**.

Variable		B	SE	β	t	*p *value	95% CI		VIF
							LL	UL	
cfPWV	Constant	–6.648	1.129		–5.890	0.000	–8.863	–4.433	
	Age	0.081	0.004	0.504	20.589	0.000	0.073	0.089	1.173
	pMAP	0.053	0.004	0.343	14.282	0.000	0.045	0.060	1.130
	FBG	0.114	0.026	0.102	4.346	0.000	0.062	0.165	1.071
	HR	0.018	0.004	0.093	4.013	0.000	0.009	0.027	1.063
	Height	0.017	0.006	0.071	2.942	0.003	0.006	0.029	1.135
	Antihypertensive treatment	0.220	0.099	0.052	2.222	0.026	0.026	0.415	1.072
	BMI	0.027	0.013	0.053	2.181	0.029	0.003	0.052	1.172
ED	Constant	438.901	6.736		65.158	0.000	425.684	452.118	
	HR	–1.620	0.054	–0.644	–30.024	0.000	–1.726	–1.514	1.034
	Sex	8.700	1.227	0.159	7.090	0.000	6.292	11.107	1.123
	BMI	–0.425	0.152	–0.063	–2.799	0.005	–0.722	–0.127	1.148
	HDL-c	5.632	1.835	0.068	3.070	0.002	2.032	9.232	1.119
	pMAP	–0.123	0.045	–0.062	–2.750	0.006	–0.211	–0.035	1.130
	FBG	–0.646	0.315	–0.044	–2.052	0.040	–1.263	–0.028	1.044

Risk factors of carotid-femoral pulse wave velocity (cfPWV) and ejection duration (ED) were analysed by multivariable linear regression analysis. Only variables that remained statistically significant in the final model were presented. pMAP, peripheral mean arterial pressure; FBG, fasting blood glucose; HR, heart rate (beats per minute); BMI, body mass index; HDL-c, high-density lipoprotein cholesterol; CI, confidence interval; LL, lower limit; UL, upper limit; VIF, variance inflation factor.

### 3.4 Multivariate Stepwise Linear Regression Analysis of cfPWV and/or ED with TOD

When cfPWV and ED were added separately to the multivariate stepwise linear regression model, cfPWV was found to be positively correlated with LVMI (0.131 ± 0.558, *p =* 0.002) and LogACR (0.123 ± 0.008, *p =* 0.004), whereas ED was negatively correlated with LVMI (–0.244 ± 0.045,* p <* 0.001) and positively correlated with eGFR(0.115 ± 0.024, *p = *0.003). This was after adjustment for age, sex, height, BMI, smoking history (yes or no), antihypertensive treatment (yes or no), HDL-c, LDL-c, FBG, pMAP and HR. These correlations did not change when both cfPWV and ED were analyzed together in the same multivariate stepwise linear regression model with the risk factors (Table 4).

**Table 4.** **Multivariate stepwise linear regression analysis of the relationships between cfPWV and/or ED with TOD**.

Variable	Covariates + cfPWV		Covariates + ED		Covariates + cfPWV and ED			
	cfPWV (β ± SE)	*p* value	ED (β ± SE)	*p* value	cfPWV (β ± SE)	*p* value	ED (β ± SE)	*p* value
LVMI	0.131 ± 0.558	0.002	–0.244 ± 0.045	<0.001	0.131 ± 0.547	0.002	–0.239 ± 0.045	<0.001
eGFR	NA		0.115 ± 0.024	0.003	NA		0.115 ± 0.024	0.003
LogACR	0.123 ± 0.008	0.004	NA		0.123 ± 0.008	0.004	NA	
CIMT	NA		NA		NA		NA	

cfPWV, carotid-femoral pulse wave velocity; ED, ejection duration; TOD, target organ damage; LVMI, left ventricular myopathy index; eGFR, estimated glomerular filtration rate; ACR, albumin–creatinine ratio; CIMT, carotid intima-media thickness; NA, none. All variables were adjusted for age, sex (male or female), height, body mass index, smoking history (yes or no), antihypertensive drugs (yes or no), high-density lipoprotein cholesterol, low-density lipoprotein cholesterol, fasting blood glucose, peripheral mean arterial pressure; heart rate (beats per minute).

### 3.5 Multivariate Logistic Regression Analysis of the Relationships between cfPWV and/or ED and TOD

When cfPWV and ED were evaluated separately by multivariate logistic regression analysis and after adjusting for covariates, cfPWV was found to be significantly associated with CKD (OR = 1.240, 95% CI 1.055–1.458, *p =* 0.009 < 0.05), while ED was significantly associated with LVH (OR = 0.983, 95% CI 0.975–0.992,* p <* 0.001). When both cfPWV and ED, together with the covariates, were analyzed in the same logistic regression analysis model, the significant associations between cfPWV and CKD, and between ED and LVH remained the same (Table 5).

**Table 5.** **Multivariate stepwise logistic regression analysis of the relationships between cfPWV and/or ED with TOD**.

Variable			B	SE	Wals	df	*p* value	OR	95% CI	
									LL	UL
Covariates + cfPWV										
	CKD	cfPWV	0.215	0.083	6.806	1.000	0.009	1.240	1.055	1.458
Covariates + ED										
	LVH	ED	–0.017	0.005	14.005	1.000	0.000	0.983	0.975	0.992
Covariates + cfPWV + ED										
	LVH	ED	–0.017	0.005	14.005	1.000	0.000	0.983	0.975	0.992
	CKD	cfPWV	0.215	0.083	6.806	1.000	0.009	1.240	1.055	1.458

cfPWV, carotid-femoral pulse wave velocity; ED, ejection duration; TOD, target organ damage; CKD, chronic kidney disease; LVH, left ventricular hypertrophy; OR, odds ratio; CI, confidence interval; LL, lower limit; UL, upper limit. All variables were adjusted for age, sex (male or female), height, body mass index, smoking history, antihypertensive drugs (yes or no), high-density lipoprotein cholesterol, low-density lipoprotein cholesterol, fasting blood glucose, peripheral mean arterial pressure; heart rate (beats per minute).

### 3.6 Multiple Stepwise Logistic Regression Analysis to Evaluate the Risk of TOD in Different Groups Defined by the Status of cfPWV and ED

After adjusting for the covariates of age, sex, height, BMI, smoking history (yes or no), antihypertensive drugs (yes or no), HDL-c, LDL-c, FBG, pMAP and HR, LVH was found to be significantly greater in Group (high cfPWV, low ED) (OR = 6.799, 95% CI 1.305–35.427, *p =* 0.023), but significantly lower in Group (high ED) (OR = 0.574, 95% CI 0.374–0.882,* p =* 0.011) compared with Group (control). However, eGFR abnormality, ACR abnormality and CIMT thickness showed no significant differences between the different groups defined by cfPWV and ED levels (Table 6).

**Table 6.** **Multiple stepwise logistic regression analysis to evaluate the risk of TOD in different groups defined by the status of cfPWV and ED**.

Variable		B	SE	Wals	df	*p* value	OR	95% CI	
								LL	UL
LVH	Age	0.051	0.008	43.228	1.000	0.000	1.052	1.036	1.068
	Sex	1.054	0.195	29.344	1.000	0.000	2.869	1.959	4.201
	BMI	0.049	0.023	4.426	1.000	0.035	1.050	1.003	1.099
	HDL-c	–0.658	0.322	4.181	1.000	0.041	0.518	0.276	0.973
	pMAP	0.020	0.007	8.206	1.000	0.004	1.020	1.006	1.034
	HR	–0.046	0.011	17.716	1.000	0.000	0.955	0.935	0.976
	Group (control)			15.464	5.000	0.009			
	Group (low ED)	0.728	0.406	3.219	1.000	0.073	2.071	0.935	4.588
	Group (high ED)	–0.555	0.219	6.420	1.000	0.011	0.574	0.374	0.882
	Group (high PWV)	0.234	0.332	0.496	1.000	0.481	1.263	0.659	2.422
	Group (high PWV low ED)	1.917	0.842	5.180	1.000	0.023	6.799	1.305	35.427
	Group (high PWV high ED)	–0.016	0.349	0.002	1.000	0.963	0.984	0.497	1.950
CKD	Age	0.073	0.016	19.393	1.000	0.000	1.075	1.041	1.111
ACR abnormality	Antihypertensive treatment	0.754	0.222	11.490	1.000	0.001	2.126	1.375	3.288
	FBG	0.145	0.047	9.392	1.000	0.002	1.157	1.054	1.269
	pMAP	0.038	0.008	20.506	1.000	0.000	1.039	1.022	1.056
CIMT abnormality	Age	0.085	0.008	102.612	1.000	0.000	1.089	1.071	1.107
	Sex	–0.696	0.189	13.499	1.000	0.000	0.499	0.344	0.723
	BMI	–0.062	0.025	6.296	1.000	0.012	0.940	0.896	0.987
	Antihypertensive treatment	0.582	0.176	10.970	1.000	0.001	1.789	1.268	2.524
	FBG	0.114	0.056	4.183	1.000	0.041	1.121	1.005	1.250
	pMAP	0.016	0.007	4.980	1.000	0.026	1.016	1.002	1.030

TOD, target organ damage; cfPWV, carotid-femoral pulse wave velocity; ED, ejection duration; LVH, left ventricular hypertrophy; CKD, chronic kidney disease; ACR, albumin–creatinine ratio; CIMT, carotid intima-media thickness; BMI, body mass index; HDL-c, high-density lipoprotein cholesterol; pMAP, peripheral mean arterial pressure; HR, heart rate (beats per minute); FBG, fasting blood glucose; OR, odds ratio; CI, confidence interval; LL, lower limit; UL, upper limit. All variables were adjusted for age, sex (male or female), height, BMI, smoking history, antihypertensive drugs (yes or no), HDL-c, low-density lipoprotein cholesterol, FBG, pMAP, HR.

## 4. Discussion

The aim of this study was to investigate the correlations of cfPWV and ED with TOD, so as to inform the possible clinical application of ED in general patients. Whether analyzed separately or together in regression models, we found that cfPWV and ED were associated with specific TOD. Multivariate stepwise linear regression showed that cfPWV was positively correlated with LVMI and LogACR, whereas ED was negatively correlated with LVMI and positively correlated with eGFR. This was observed regardless of whether cfPWV and ED were analyzed separately or in combination. Multivariate stepwise logistic regression analysis showed that cfPWV was only associated with eGFR abnormality, whereas ED was associated with LVH after adjusting for covariates and when analyzed either individually or in combination with cfPWV. The association of ED with LVH was statistically significant when cfPWV was in the normal range. With low ED, elevated cfPWV appeared to significantly affect LVH.

In this study, cfPWV and ED were entered either separately or together into the regression models with the covariates. Since both cfPWV and ED were generated by pulse wave analysis, they were entered separately into the regression models to avoid multicollinearity. However, both cfPWV and ED were associated with TOD, hence they were entered into the same regression model with the covariates in order to evaluate and compare their impacts on TOD. The TOD in different patient groups defined by cfPWV and ED status was also analyzed in this study to help elucidate the possible interactions of cfPWV and ED with TOD. With this approach, the associations of cfPWV and/or ED with TOD could be comprehensively assessed.

The results of this study suggest that the observed association between cfPWV with renal damage was the same using either multivariate stepwise linear or logistic regression analysis, whereas the association between cfPWV and LVMI was not. In contrast, ED was correlated with LVMI and eGFR by multivariate stepwise linear regression analysis, but was only associated with LVH by multivariate stepwise logistic regression analysis. It has been reported in earlier studies that cfPWV was associated with cardiovascular events and TOD [3, 27, 28, 29]. In our previous study, cfPWV showed a significant negative association with eGFR, and the association between arterial stiffness and CKD suggested that cfPWV may be a potential hemodynamic index to evaluate cardiovascular risk in CKD patients with primary hypertension [30]. Moreover, a review of arterial stiffness and CKD reported that pulse wave velocity in patients with CKD is much higher in those with diabetes compared to patients of similar age but without diabetes [31]. The present study showed that cfPWV was correlated with LogACR and was associated with eGFR. These findings concur with previous research showing that cfPWV was significantly associated with CKD and microalbuminuria, suggesting that cfPWV is a vessel-related and renal-related biomarker [32]. However, other studies have shown that arterial stiffness correlates with albuminuria but not with mild-to-moderate CKD [33], thus indicating the need to further investigate the relationship between cfPWV and CKD. In the current study, ED was found to be associated with LVH. ED is defined as the time in the cardiac cycle during which the left ventricle actively ejects blood through the aortic valve and into the circulation [34]. ED has demonstrated value for CVD risk assessment in longitudinal studies [14] and for the progression of heart failure [35]. A proportional relationship was demonstrated between the duration of left ventricular ejection time (LVET), which is a component of systolic function, and overall external myocardial efficiency [36]. A shorter LVET is known to worsen external efficiency. LVET is also directly correlated with the left ventricular ejection fraction (LVEF) and with stroke volume. It is shortened in heart failure with reduced ejection fraction (HFrEF) [37]. In the present study, ED was negatively correlated with LVMI and also with LVH (OR <1), which is similar to previous reports [36, 37]. Regarding the positive correlation observed in the current study between ED and eGFR, Chen *et al*. [38] found that brachial pre-ejection period (bPEP)/brachial ejection time (bET) was an independent determinant of LVMI and LVEF and was helpful for the prediction of LVEF in patients with CKD. Therefore, the relationship between ED and eGFR requires further clarification.

After adjusting for covariates, we found that LVH was significantly higher in Group (high cfPWV, low ED) patients, but significantly lower in Group (high ED) patients. Previous studies have reported an association between arterial stiffness and left ventricular systolic function [39, 40]. In the present study, shorter ED and elevated cfPWV increased the risk of LVH, whereas normal cfPWV and increased ED was correlated with a significantly lower risk of LVH. Increased cfPWV suggests an increase in arterial stiffness, thereby contributing to dysfunction of cardiac systolic function and thus affecting ED. A previous study showed that cfPWV was significantly associated with LVH in CKD patients [39]. Central PP (pulse pressure), Aix and aortic PWV are key measures of arterial function and are susceptible to left ventricular performance [40]. ED is reported in milliseconds (ED ms) and as a percentage of the cardiac cycle (ED%). Biering-Sørensen *et al*. [14] found that a shorter LVET (ED ms) was associated with younger age, male sex, higher diastolic blood pressure (BP), higher incidence of diabetes, higher heart rate, higher blood glucose levels and worse fractional shortening (FS), while a lower LVET (ED%) was associated with a significantly increased risk for all events. Although the interactions between ED in combination with cfPWV and LVH are still unverified, the present study suggests there may be dependent or independent associations between ED and arterial stiffness with LVH. This requires further research before individualized management of patients can be achieved.

This study has several potential limitations. Due to its cross-sectional study design and relatively small sample size, the results need further verification in prospective studies. The study was conducted in an Asian population, and hence it is not known whether the results also apply to other ethnic groups. Furthermore, the associations between cfPWV and/or ED with TOD were studied in a general population sample, and comparison of genders should be further investigated. Despite the statistical differences observed for the interactions of cfPWV and ED with LVH, the intrinsic mechanisms involved require further investigation. Although the effects of ED on TOD were discussed in this study, the relationships between pre-ejection period (PEP)/LVET, cardiovascular outcomes and TOD remain unexplored and should be investigated in future studies. The ankle-brachial index (ABI) is defined as the ratio of systolic blood pressure between the ankle and the arm [41]. The ABI is of great significance in screening for peripheral artery disease (PAD) and for predicting cardiovascular disease [42, 43]. A low ABI is an indicator of atherosclerosis, and cfPWV is known to increase as arterial stiffness increases. In an elderly Chinese cohort, the upstroke time per cardiac cycle in the lower extremities showed a significantly stronger association with vascular and renal damage compared with the ABI [44]. Although ABI was not evaluated in the current study, the pathophysiological associations between ABI, cfPWV and ED warrant further research. Finally, the subendocardial viability ratio (SEVR) is an index of myocardial oxygen supply and demand that can be evaluated noninvasively using applanation tonometry. Low SEVR has been associated with reduced coronary flow reserve in patients with hypertension [45]. Although in the current study SEVR was compared between males and females, our focus was on the interaction between cfPWV and ED. Further studies on SEVR should therefore be considered in future research.

## 5. Conclusions

In conclusion, cfPWV was more strongly associated with renal damage, whereas ED was more strongly associated with LVH. cfPWV and ED affect each other and have a combined effect on LVH. Clinically, more attention should be paid to LVH in patients with high cfPWV and low ED. However, patients with low cfPWV and high ED are likely to have a lower risk of LVH.

## Data Availability

The datasets used and/or analyzed during the current study are available from the corresponding author on reasonable request.

## Abbreviations

cfPWV, carotid-femoral pulse wave velocity; ED, ejection duration; TOD, target organ damage; LVMI, left ventricular mass index; ACR, albumin-creatinine ratio; eGFR, estimated glomerular filtration rate; CKD, chronic kidney disease; LVH, left ventricular hypertrophy; CIMT, carotid intima-medium thickness; BMI, body mass index; UA, uric acid; Cr, creatinine; TC, total cholesterol; LDL-c, low-density lipoprotein cholesterol; HDL-c, high-density lipoprotein cholesterol; FBG, fasting blood glucose; HbA1c, hemoglobinA1c; cSBP, central systolic blood pressure; cDBP, central diastolic blood pressure; cMAP, central mean arterial blood pressure; cAIx, central augmentation index; cAIx@HR75, cAIx adjusted to heart rate of 75 bpm (beats per minute); SEVR, subendocardial viability ratio; pMAP, peripheral mean arterial blood pressure; K/DOQI, Kidney Disease Outcomes Quality Initiative; MDRD, Modification of Diet in Renal Disease; HR, heart rate; IMT, intima-Media Thickness; TG, total triglycerides; pDBP, peripheral diastolic blood pressure; cDBP, central diastolic blood pressure; cMAP, central mean arterial blood pressure; pSBP, peripheral systolic blood pressure; cAP, central augmentation pressure; CVD, cardiovascular disease; LVET, left ventricular ejection time; LVEF, left ventricular ejection fraction; HFrEF, heart failure with reduced ejection fraction; bPEP, brachial pre-ejection period; bET, brachial ejection time; BP, blood pressure; FS, fractional shortening; PEP, pre-ejection period.
